# Curious Effects of Roman Air

**Published:** 1831

**Authors:** 


### LXXIII.

### Curious Effects of Roman Air.

Although the rage for Italian residence and Italian air is probably much on the decline, yet the attractions of that classic soil are such that many people make HEALTH the excuse for visiting that fairy land. If these should suffer for their curiosity, no very great share of pity can be extended to them. But there is a large class of REAL INVALIDS who consult their medical attendants on the propriety of trying the air of Italy for relief of their sufferings, and their medical advisers are often placed in awkward positions on these occasions. If they dissuade their patients from the trial, they are sometimes suspected of self-interest—if they advise them to proceed, they may be adding to the evil already incurred. The safe course for the medical practitioner is to advert to the sentiments of those who have investigated the subject personally. There is much to be gained from the experience of invalids themselves; and a selection from their observations alone, would be a very useful little volume for those medical practitioners in this country, who are unable to speak from personal experience. Matthews, in his Diary of an Invalid, has made many shrewd remarks on this subject, and so has the late Mr. Hope, in the novel of Anastasius. But we cannot pursue this subject further on the present occasion. Of the medical authorities respecting the climate of Italy, Dr. Clark holds, of course, a distinguished place, and he is an excellent authority to refer to, on most occasions. The second edition of his work is evidently less favourable to an Italian climate than the first—no doubt, from more matured experience and multiplied observation. The following quotation from a recent tour through Italy may not be uninteresting at this season, when invalids are pluming their wings for a flight to southern regions.

### Nervous Disorders—Roman Sensibility.

"Under this vague term (nervous disorders) a host of dissimilar and really different maladies is comprehended. There is no doubt that a *journey* to Rome would generally be beneficial to people affected with nervous complaints; but it is very questionable if a residence there would be productive of substantial good. It is a remarkable fact that the inhabitants of the Eternal City are characterized by a peculiar sensibility of the nervous system—evinced by a disposition to convulsive affections, from causes quite inadequate to the production of such phenomena in other people and in other countries. The inordinate sensitiveness of the Roman ladies to perfumes is well known, and might be almost taken for freaks of the fancy, were it not so well authenticated. It is a susceptibility, too, of recent origin. The Roman matrons of old were fond of perfumes—those of the present day often faint, or go into convulsions, on perceiving the odour of the most pleasant flower. And not females only, but effeminate males evince the same morbid sensibility to odoriferous emanations.* The causes of this phenome-

---

* "Dr. Mattæi (whom I had the pleasure of knowing in Rome) states, in non have given rise to diversity of opinions. The Roman physician (Mattæi) attributes it to 'the daily increasing mobility of the nervous system, produced by the luxurious and listless life of the Roman people.'† But Dr. Clark, while he admits that such a life may have tended to originate this morbid sensibility, and that, when once acquired, it may be transmitted from parent to progeny—believes that '*the climate of Rome has some specific effect in inducing this state of the nervous system.*' I have no doubt of it. And my only wonder is, that Dr. Clark, during ten years' residence there, did not find out what this something is. He says, in the same page:—'Even a temporary residence of some duration at Rome, produces a degree of the same morbid sensibility, and, in cases where the Roman mode of living cannot be adduced as the cause.' I think I hear the reader ask, what is this cause, then, which has so much puzzled the doctors? If compelled to answer, I would say that it is the habituation to the STINK of the Roman streets, which perverts the sensibilities of the olfactory nerves—renders them unaccustomed to decent smells—and throws them into convulsions on contact with a perfume. I accord entirely with Mr. Matthews, in the opinion that the former MISTRESS of the WORLD is now the dirtiest city in Europe—with the exception of Lisbon. This solution explains another part of the phenomenon which puzzles Dr. Clark. 'It is to be remarked, (says he,) that it is not *disagreeable odours* which produce such effects on the nervous system, but the more *delicate*, and, to northern nations, *agreeable odours* of flowers and other perfumes.' No doubt of it. If mal-odorous exhalations had been capable of inducing convulsions, Rome would, long since, have cured the evil effectually, by removing from the presence of her insulted ruins, the cause of it—MAN!"‡

"But there is another and a much more formidable malady, or rather class of maladies, to which the Romans are peculiarly prone—namely, *sudden death*, —or, as it is coolly called, ACCIDENTE—which is sometimes sporadic, sometimes EPIDEMIC in Rome.§ Whether this terrific agent of the Grim Tyrant acts through the medium of apoplexy or diseases of the heart, the Roman physicians have not ascertained—but one thing is clear, that the climate of the Eternal City is extremely hostile to the brain and nervous system—and consequently all who have any tendency to fulness about the head should be shy of residence there. Dr. Clark observes that—'head-aches are common at Rome, and, among strangers, he has found them of very frequent occurrence.' The same author, however, informs us that *bronchial affections* (chronic inflammation of the mucous membrane of the air-tubes) are generally benefited by a Winter's residence in Rome—as also chronic rheumatism. But the passage which I have already quoted, some pages back, from Dr. Clark, respecting the frequency and severity of inflammatory affections of the chest, during Winter and Spring, in Rome, casts strong doubts on this utility of the climate in CHRONIC BRONCHITIS. That the Italian winds, like the satyr's breath, blow hot and cold, almost at the same moment,

---

his clinical work, as follows:—'Nostra vero ætate nervosæ affectiones, vulgo *tirature*, seu convulsiones *communissimæ* sunt, fæminis presertim, *effeminatisque viris*, quorum corpora a tam levibus causis commoveri solent, ut odorum licet gratissimorum vis ea facile perturbet ac male afficiet.'"

† "A molli inertique vita in Romanis incolis."

‡ What says the eloquent author of ANASTASIUS?

"These people (the modern Romans) cannot prevent the sun of their fine climate from shining at its stated hours; but they make their streets impervious to its cheerful light. They cannot prohibit the rich vegetation of their fertile soil from diffusing its fragrance; but they collect every villainous odour to subdue Nature's sweets. *Even amid their orange-groves, loss of scent would be gain.*"

§ "Subitanea scilicet mors, *vulgo* ACCIDENTE, quæ a diversis causis ortum ducens, modo sporadica, modo quasi epidemica obrepit."—*Mattæi.*

I am ready to grant; but, in a strictly medical sense, I leave my talented friend to explain how a climate, in which 'acute inflammation of the lungs appeared more violent and more rapid in its course than in England,' can possess the singular and felicitous property of relieving already existing inflammation of the tubes leading to the same organ. I bow to his authority, as to the *fact*—I only state the difficulty of the *explanation*. But I shall conclude the subject of apoplexy and nervous affections, with the following short and apposite sentence from the same author.

'For persons disposed to apoplexy or nervous diseases, Rome, of course, would not be selected as a residence—nor is it proper for persons disposed to *hæmorrhagic* diseases—or for those who have suffered from intermittent fevers.'

I need hardly say that hæmorrhage, or bleeding from the lungs, is one of the most common precursors, causes, and accompaniments of pulmonary consumption;—and this fact, taken in conjunction with all that has been offered respecting the climate of Rome—one of the most favourable of the Italian climates for consumption—ought to inspire serious doubts as to the propriety of directing phthisical invalids to the Eternal City—unless it be for the purpose of enjoying eternal repose near the pyramid of Caius Cestius."*—*Johnson on Change of Air.*

* "The English burial ground—where a fosse or ditch, instead of a wall, surrounds and protects those 'frail memorials' of our departed countrymen, which—

'Implore the passing tribute of a sigh,'

from every one who has a spark of feeling in the heart."—*See View from the Tower of the Capitol, p.* 160.

---

